# Fostering formative assessment: teachers’ perception, practice and challenges of implementation in four Sudanese medical schools, a mixed-method study

**DOI:** 10.1186/s12909-023-04214-3

**Published:** 2023-04-14

**Authors:** Elaf Abdulla Almahal, Abrar Abdalfattah Ahmed Osman, Mohamed Elnajid Tahir, Hamdan Zaki Hamdan, Arwa Yahya Gaddal, Omer Tagelsir Abdall Alkhidir, Hosam Eldeen Elsadig Gasmalla

**Affiliations:** 1 Faculty of Medicine, Al-Neelain University, Khartoum, Sudan; 2 Department of Human Physiology, AlMughtaribeen University, Khartoum, Sudan; 3 Biochemistry Department, Faculty of Medicine, Al‑Neelain University, Khartoum, Sudan; 4 Department of Basic Medical Sciences, Unaizah College of Medicine and Medical Sciences, Qassim University, Unaizah, Saudi Arabia; 5 Anatomy Department, University of Medical Science and Technology UMST, Khartoum, Sudan; 6 Anatomy Department, Faculty of Medicine, Al-Neelain University, Khartoum, Sudan; 7 Clinical Anatomy and Imaging, Warwick Medical School, University of Warwick, Coventry, United Kingdom

**Keywords:** Students, Assessment, Formative assessment, Assessment for learning, Challenges

## Abstract

Formative assessment (assessment for learning) enhances learning (especially deep learning) by using feedback as a central tool. However, implementing it properly faces many challenges. We aimed to describe the perception of medical teachers towards FA, their practice, challenges of implementing FA and present applicable solutions. A mixed-method, explanatory approach study was applied by administering a validated questionnaire to 190 medical teachers in four medical schools in Sudan. The obtained results were further studied using the Delphi method. Quantitative analysis revealed that medical teachers perceived their grasping of the concept of FAs and their ability to differentiate formative from summative assessments as very well (83.7%) and (77.4%), respectively. However, in contradiction to the former results, it was noteworthy that (41%) of them mistakenly perceived FA as an approach conducted for purposes of grading and certification. The qualitative study defined the challenges into two main themes: lack of understanding of formative assessment and lack of resources. Medical teachers’ development and resource allocation were the main recommendations. We conclude that there is misunderstanding and malpractice in implementing formative assessment attributed to the lack of understanding of FA as well as the lack of resources. We as well present suggested solutions derived from the perception of the medical teachers in the study and evolved around three approaches: faculty development, managing the curriculum by allocating time and resources for FA, and advocacy among stakeholders.

**Supplementary Information:**

The online version contains supplementary material available at 10.1186/s12909-023-04214-3.

## Introduction

Enhancing learning using feedback as a central tool of formative assessment (FA) is of paramount importance [1]. However, implementing formative assessment appropriately in developing countries such as Sudan faces challenges. Medical education in Sudan can be traced from 1924 when the first medical schools were established, during the 1970s, two other medical schools were added. In the last 30 years between the 1990s and 2022, the number of medical schools increased rapidly to more than 70 medical schools. Medical education in Sudan has been enhanced mainly because the different stakeholders (academics and policymakers) have been working on addressing the challenges. In 2018, Sudan Medical Council (established in 1955) was awarded recognition status by the World Federation of Medical Education. Making it the first accrediting body among the Arabian countries and the tenth worldwide [2, 3]. Identifying the challenges of implementing FA is the first step towards formulating an approach to resolve them and would positively impact the growing number of medical schools in the country. Guidelines in medical education are context-dependent; thus, this work will add to the Sudanese library that addresses national issues in education based on local cultural context. This study applied a mixed-method, triangulation approach to investigate the perception of 190 medical teachers across four medical schools in Sudan towards FA, their practice, their perceived challenges of implementing FA and present applicable solutions.

## Literature review

FA definition, principles, purposes and practices: FA has been considered a distinguishing entity from summative assessment [1, 4]. Its notion has shifted from the context of programme evaluation to being based on the benefits of the learners. It can be seen as a process performed during learning rather than a test at the end of the course [5]. The system of assessment is based on the balance between both summative and FAs [6]. It is the features of formative assessment that provide its benefits. Using feedback as a central tool, information about performance and competencies is collected to facilitate learning (especially deep learning) [5, 7]. It motivates the students [8] and shifts their minds from focusing on just obtaining high grades on the final exam to engagement in learning and skills development [9]. Moreover, FA promotes learning outcomes by creating communication between the learner and the teacher via feedback [10]. Feedback is considered a keystone in FA [11] since feedback aims to support learners to achieve learning outcomes. It is a way to inform the learner about the gap between his/her current status and the learning outcomes, a comparison between the performance of the learner and the standard [12]. This comparison to the standard (a criterion) makes FA fitting for the criterion-referenced approach of standard setting [13], since it. The importance of feedback is paramount, generally, better performance in FAs is associated with better performance in the final exam, as reported by Krasne, Wimmers [8] and McNulty, Espiritu [14]. It must be noted, however, that even students who did not receive scores for success in FA were able to benefit from feedback that was useful in helping them succeed in their final examinations. This was primarily the result of their active participation in FA and the use of the feedback that they received during that time [5, 11, 15]. Furthermore, FA is a continuous process in which feedback is not the final element, but a continuous component that identifies improvements in the learner's performance [16]. Thus, FA is seen as one of the features of tomorrow’s education that is based on assessment for learning [4].

In this study, the term FA is used to indicate a process that is an assessment for learning, conducted in class, not judgmental, in which feedback is provided, and it is not taking part in the final summative assessment.

Challenges of implementing FA were investigated. The lack of FA implementation is considered a deficiency in medical education practice [17]. Nevertheless, it is attributed to the way FA is perceived by both medical teachers. The lack of comprehension of the concept of FA and its value [18] leads to resistance possibly attributed to perceptions driven by the educational traditions in the clinical setting [17]. There is a positive correlation between the awareness and perception of medical teachers toward FA and its application [19]. The lack of awareness about feedback as an important tool in FA was reported to be a challenge that creates a gap between students' expectations and teachers' perceptions [20]. Giving feedback to a diverse set of students was perceived to be potentially challenging with different cultures and languages especially if the feedback contained negative elements [21]. Aside from the lack of awareness, challenges such as time and resource constraints were spotted [19]. These constraints were sometimes manifested as difficulties in finding time for preparation, and the overcrowded schedules of the staff as well as the students which in turn affected their commitment to FA [22].

This study aimed to describe the perception of medical teachers towards FA, their practice, the challenges they face in implementing FA and their suggested solutions.

## Methods

This was a cross-sectional, mixed-method study. The study consisted of two phases. Phase one was quantitative and data collected this way were further investigated qualitatively in phase two, this is an explanatory approach to mixed-method studies [23]. In the quantitative phase data about the perception and current practices of the teacher regarding FA were collected using a questionnaire. Then, based on the responses to the questionnaire, further identification of challenges in the implementation of FA and recommended solutions was conducted using the Delphi technique, a qualitative data collection tool. For ease of description, each section below is denoted whether it applies to phase one, two, or both phases.

### Setting and context (for both phases – Fig. 1)

**Fig. 1:**
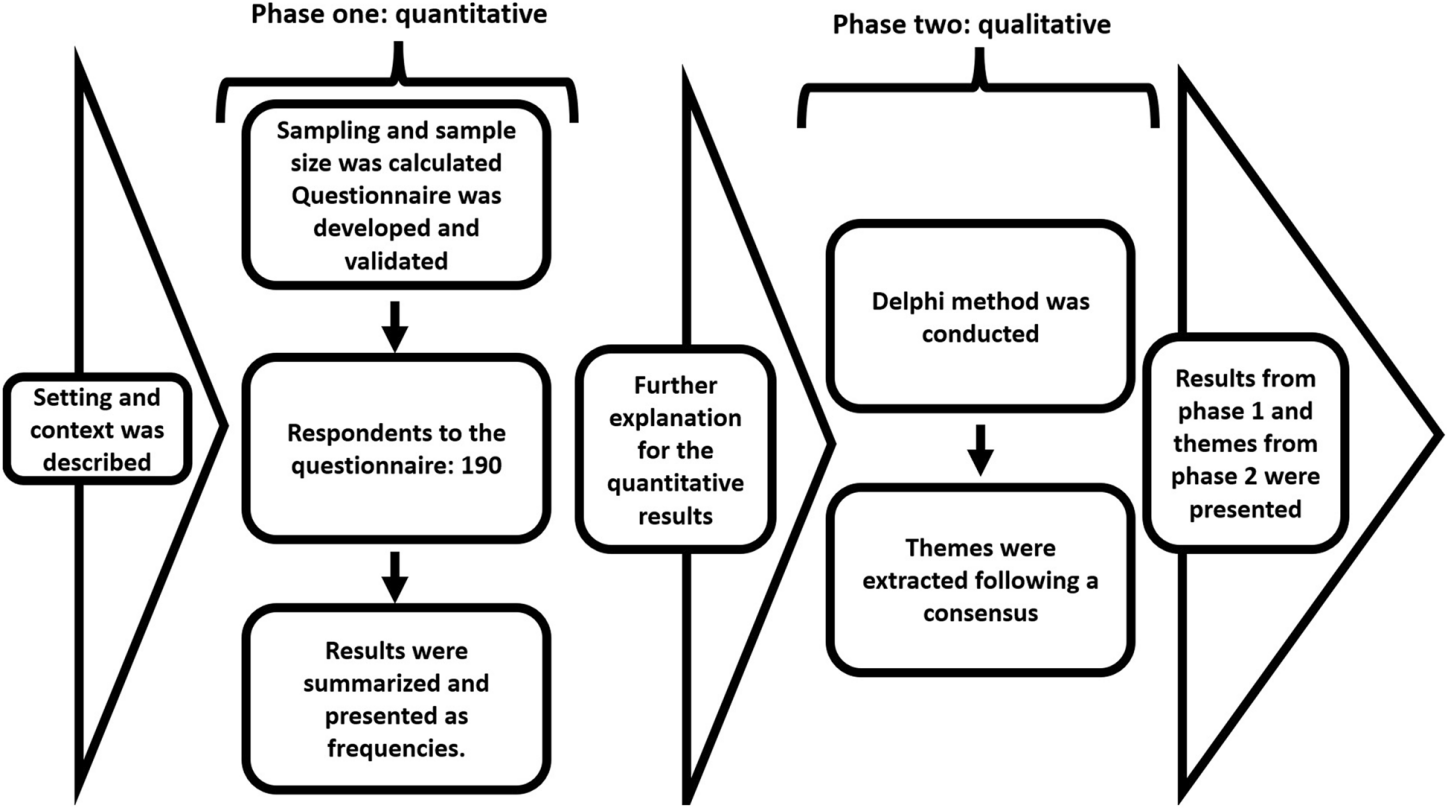
Explanatory mixed-method

For both phases of the study, the selection of medical schools was based on several criteria, first, the medical school must be among the largest medical schools in Sudan in terms of the number of medical teachers. Second, the selected medical schools must represent both public and private sectors, and third, the selected medical schools must represent both classical and integrated curricula. These criteria were followed to ensure representativeness and reflect all the diversities among medical schools in the country. To this end, we selected four medical schools, each one with no less than 200 students/batch and full-time staff ranging from 55 to 120. Two schools represent the public and private sectors. One of the public schools adopts a classic curriculum while the other one adopts an integrated curriculum, the same goes for the two private schools. This combination of (public/private) and (classical/integrated curricula) was intended to include and represent all the varieties in Sudanese medical schools.

### Phase one: quantitative study

In this phase, the sample size was calculated, and a questionnaire was developed and sent to a sample of medical teachers.

### Sampling and sample size (for phase one)

The population are teachers at four medical schools. Inclusion criteria include all full-time appointed teachers who are ranked from the lecturer and above, teaching assistants are excluded since they are not allowed to develop and conduct assessments.

The sample size of 165 was calculated by an online Open-epi calculator, based on the predicted anticipated subjects. After we estimated the eligible population in the four universities, we assumed that 50% of the medical teachers will participate in this phase of the study. This would give the study an 95% power to detect differences of 0.05 at the α-level [24].

### Questionnaire development and validation (for phase one)

A questionnaire (see appendix) was developed following a thorough search in the literature; the questionnaire consists of 22 questions covering three domains: demographic data, perception of FA, and practice. The first domain consisted of 4 questions about gender, job title/rank, years of experience (in teaching) and the department, the second domain consisted of statements about the perception of FA, with a five-point Likert scale (5 = strongly agree, 4 = agree, 3 = I am not sure, 2 = disagree, 1 = strongly disagree) while the third section consisted of statements about the practice of medical teachers concerning FA, with three-points Likert scale (3 = never, 2 = sometimes, 1 = regularly).

The questionnaire was in English since medical education in Sudan is in the English language. It was tested in a pilot study, in which three experts (minimum ranking of assistant professors, with experience in health professions education, questionnaires development and validation and at least 10 years experience in teaching and student assessment) first reviewed it to validate the contents, minor changes were applied following their recommendations, the changes focused on simplifying the language of the questionnaire by replacing some sophisticated terms by simpler ones. Then it was introduced to 33 university teachers to investigate its face validity, practicality and reliability [25, 26]. Cronbach’s alpha was calculated. It was 0.63 for questions regarding the perception and 0.55 for questions regarding the practice.

The questionnaire was then sent out via a google form link to the medical teachers in the targeted medical schools.

### Phase two: qualitative study: the delphi technique

The Delphi technique is a qualitative approach to reaching a consensus. It consists of an iterative process [27].

Aim: The technique aimed to answer two questions: a) What are the challenges of implementing FA in your setting? b) What are the suggested solutions? The questions were constructed after a systematic search in the literature using the search terms (challenges/difficulties of implementing/ implementation/ of FA and fostering/enhancing FA). The search was conducted in PubMed and Scopus during the period from January to May 2020 and included all types of articles in the English language published since 2000.

Participants (in phase two): a group of six university teachers was invited, the recruitment of participants followed a nonprobability purposive sampling, and the group consisted of assistant professors who were experts in the field of medical education (including a master's degree as a minimum, active participation in the education development units and with publications in medical education), with experience in university teaching not less than five years, they represented the basic medical sciences and clinical sciences departments.

Validity and reliability: the two questions were piloted in a group of five experts in medical education. With experience in university teaching ranging between 5 to 10 years. The purpose was to ensure the clarity and simplicity of the wording, regarding reliability, the group of participants consisted of six, and the recommended number of participants that ensures reliability is ranging between 6 to 12 [28] with some authors referring to 7 participants as a minimum [29].

Ethical issues and anonymization: participants gave their written consents before participation, they were not aware of the identities of each other; however, they were known to the authors. The results of the study were not affecting the participants. Hence there was no conflict of interest, and to our best knowledge, no participants' bias was noted.

Informing the participants: written information was provided with the questionnaire in the first round describing the nature of the process, some key features of FA were written to keep the participant engaged and to avoid any confusion with the summative assessment.

Cutoff point: We agreed that the cutoff for continuation is 70% consensus [30], i.e. if 70% of statements gained consensus the study would be determined to be complete. To adapt to the hectic schedules of the participant, no deadline for the ending of each round was set.

Consensus roles: if the statement gained more than three on average on a 5-point Likert scale, then this was considered as consensus. If the score was less than three, then the statement was discarded.

Round one: consisted of open-ended questions, and there was no feedback in this stage, from the responses of round one, the authors produced statements that were put for ranking in a 5-point Likert scale and used in the successive rounds.

Consecutive rounds: the first three authors reviewed input, and topics were arranged and modified after discussions between the mentioned authors, in the successive rounds, newly introduced topics, modified topics, and topics not reaching the consensus were presented along with their statistics.

### Data analysis (for both phases)

#### Phase one: quantitative

Ordinal data obtained from the participants’ responses to the questionnaire were converted to quantitative data. The collected data were analyzed using the Statistical Package of Social Sciences (SPSS), and they were summarized and presented as frequencies.

#### Phase two: qualitative

Each statement was ranked in the next rounds. The scores were tabulated. The consensus roles were applied to determine the number of statements that reached consensus.

### Ethical approval

Was obtained from Al-Neelain University Ethics Review Board.

## Results

### Quantitative: formative assessment: perception and practice

Following the introduction of the questionnaire, the respondents were 190 medical teachers out of 288 across all four medical schools (which is a bigger number than the target sample size of 165). The average of respondents in each medical school was 66 ± 2.6% of the total targeted population in that school. Demographic data showed the percentage of males was 47.9% and females 52.1%. Assistant professors share was half the respondent and the faculty who teach basic medical sciences and clinical sciences are almost equal (Table 1).

**Table 1:** Demographic data of the faculties participated in the quantitative study

Gender	Male	47.9% (*n* = 91)
	Female	52.1% (*n* = 99)
Job title	Lecturer	33.7% (*n* = 64)
	Assistant professor	50.5% (*n* = 96)
	Associate professor/professor	15.8% (*n* = 30)
Department	Basic medical sciences	45.8% (*n* = 87)
	Clinical sciences	46.8% (*n* = 89)
	Community medicine/public health	7.4% (*n* = 14)

The study of the knowledge revealed that the medical teachers perceived their understanding of the concept of FAs (83.7%) and the difference between formative and summative assessments as very well (77.4%), they also perceived feedback as a keystone in FA (87.4%). Furthermore, they appear to realize the benefits of FA, such as it encourages deep learning, as well as the association between better performance in FAs and the final exam (Table 2). However, it was a notable contradiction that (41%) of them mistakenly perceived FA as an approach conducted for purposes of grading and certification with about 18% didn’t know whether this fact about FA is correct or not, another contradiction was that 43% agreed that the student’s final grades in a course are collected from his/her grades in the FAs (and about 18% are not sure). Also, when asked if FA is criterion-referenced, (42%) didn’t know the answer and (22%) said it is not.

**Table 2:** Formative assessment: perception of medical teachers

	Strongly agree	Agree	I am not sure	Disagree	Strongly disagree
I understand the concept of formative assessment	25.8%	57.9%	11.1%	4.2%	1.1%
I recognize the difference between formative assessment and summative assessment	27.4%	50%	14.2%	5.8%	2.6%
Formative assessment conducted for purposes of grading and certification	11.6%	30%	17.9%	27.4%	13.2%
Formative assessment encourages superficial learning	3.7%	21.1%	17.9%	47.4%	10%
Formative assessment encourages deep learning	24.7%	51.1%	16.3%	6.3%	1.6%
Formative assessment considered as criterion referenced	5.3%	39.5%	42.6%	11.1%	1.6%
Feedback is keystone in formative assessment	36.3%	51.1%	8.4%	3.2%	1.1%
Better performance in formative assessment is associated with better performance	22.1%	55.8%	11.1%	8.4%	2.6%
The students’ final grades in a course are collected from his/her grades in the formative assessments	6.3%	37.4%	17.9%	32.6%	5.8%

The study of the practice revealed that only a third of the participants regularly conduct FA and provide feedback to students in FAs. Regarding adding the scores obtained by the student in the FA to his/her final grades at the end of the course/semester/year. Third of the participants reported doing that regularly, with another third doing it sometimes (Table 3).

**Table 3:** Formative assessment: practice responses

	Never	Sometimes	Regularly
I conduct formative assessment	8.4%	59.5%	32.1%
I provide feedback to students in formative assessments	13.2%	52.6%	34.2%
I add scores obtained by the student in the formative assessment to his/her final grades of the end of the curse/semester/year	34.2%	31.6%	43.2%

**Fig. 2:**
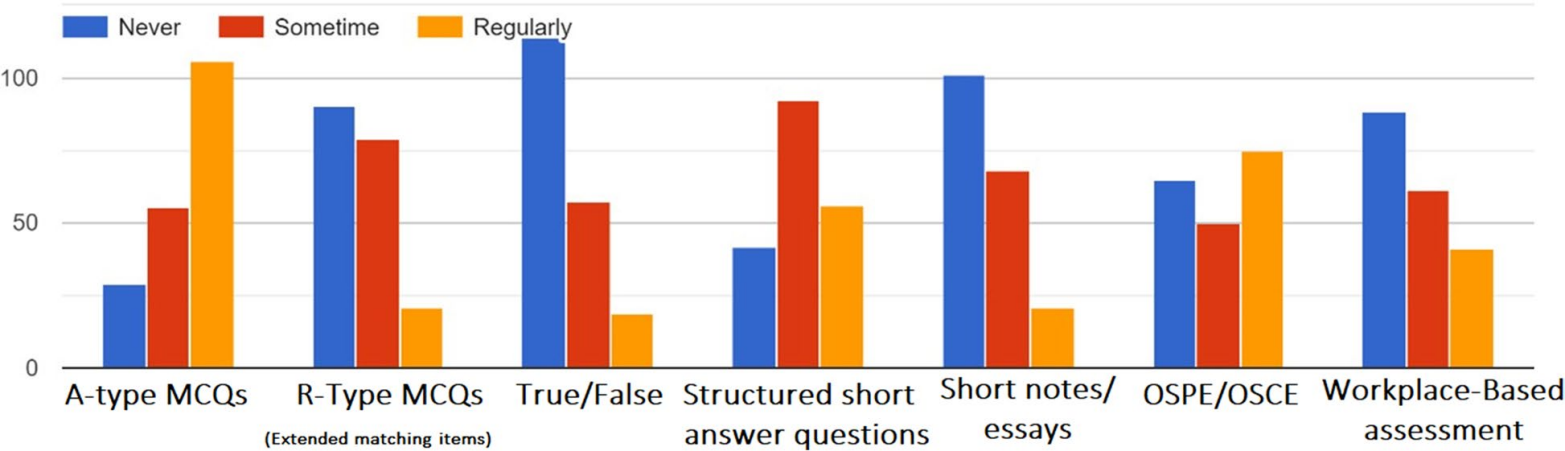
Assessment tools used in FA

The most employed assessment tool used in FA was the MCQs A-Type followed by OSCE (Fig. 2), while the least tool to be used were MCQs R-Type and the essays.

### Qualitative: challenges and suggested solutions

**Fig. 3:**
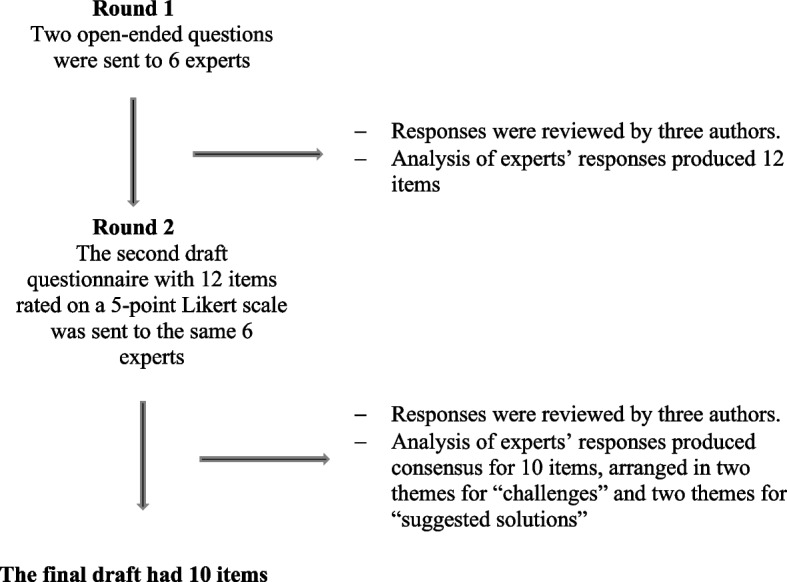
The process of Delphi

Six experts participated in the first and second Delphi rounds (Fig. 3). In the first round, two open-ended questions were sent, the first question inquired about the challenges of implementing formative assessment while the second question was about the suggested solutions.

All the Delphi panellists were senior medical teachers with a minimal ranking of assistant professors. The first round generated 12 items. Following the second round, a consensus was reached by all of the panellists and the final number of items was 10 across four main themes (Table 4). Two themes were about the challenges of implementing FA and the other two themes were about the suggested solutions.

**Table 4:** Topics and summary of the Delphi process rounds 1 and 2

Round One	Round Two	
	Items	Themes
Inadequate understanding of the role of formative assessment	Inadequate understanding of the role of formative assessment	Challenges of implementing FA: Lack of understanding
Lack of staff commitment	Lack of staff commitment	
Students don’t focus on the formative assessment because of its timing	Students don’t focus on the formative assessment because of its timing	
Lack of resources	Lack of resources	Challenges of implementing FA: Logistics and resources
Large number of students		
Lack of time	Large number of students	
Implementation of formative assessment is exhausting to the medical teachers		
Time allocation	Time allocation	Suggested solutions: resources allocation
Resources allocation	Resources allocation	
Adopt a reasonable students staff ratio	Adopt a reasonable students staff ratio	
Use of online assessment/other technologies	Use of online assessment/other technologies	
Training of staff	Training of staff	Suggested solutions: faculty development

Concerning challenges, the participants agreed on the lack of understanding as one of the two themes. They elicited an inadequate understanding of the role of formative assessment, lack of staff commitment, students don’t focus on the formative assessment because of its timing as a feature for this theme. The other theme was about logistics and resources. This includes a large number of students (per staff) and the lack of resources.

Concerning suggested solutions, the participants agreed on the lack of resource allocation as one of the two themes. They elicited time allocation, resource allocation, adoption of a reasonable student-staff ratio and the use of online assessment/other technologies as features for this theme. The other theme was faculty development. This includes the training of staff.

## Discussion

### Overview

FA enhances deep learning using feedback as a central tool. However, the benefits of FA are only gained by doing it properly and overcoming the challenges associated with its implementation. The current study investigated the perception of medical teachers about FA and their practice via quantitative analysis. The qualitative assessment is used to explain the quantitative findings: the gaps in understanding and implementing FA revealed by the quantitative analysis were further studied qualitatively, as a result, factors that contribute to the challenges in implementing FA were elaborated along with the suggested solutions.

### Perception and practice of the medical teachers: what does the quantitative analysis show?

The study of how much medical teachers know about FA revealed that while most of them believe they understand FA, the detailed further questions reveal that they don’t (Table 2). The same inconsistency was found between their perceived knowledge and their actual practice. While most of the participants acknowledge the value of FA, only a third of them conduct FA regularly (Table 3). Which raises a red flag about “what we think we know”. This inconsistency was explained when combining the qualitative data retrieved by experts since the “lack of understanding” of the concept of FA was highlighted in the Delphi study. This reveals that most of the medical teachers included in the current study mistakenly believe they comprehend the notion of FA. However, this phenomenon is not unique, and it was also noted previously [18]. Therefore, the issue of the “lack of understanding” is deeply rooted as a challenge.

Another manifestation of the lack of understanding was reported when the participants asked about the most assessment tool they use in FA. It was the MCQs A-Type (Fig. 2). While MCQs R-Type was less utilized. Both formats are objective assessment tools with a restricted response. they comprise three components: question (or stem), which can be a scenario or clinical vignettes, lead-in questions, and options. In the A-type MCQs, the options (distractors) vary between three and five but usually around four or five responses with one correct (or one best) answer. While in R-type, there can be up to 26 options [31, 32]. The R-Type MCQs are reported to be well suited for encouraging deep learning [33] and assessment of clinical reasoning [34–36], the same goes for the free-response (or open-ended) questions, which can assess the higher levels of cognitive functions and clinical reasoning [37]. The figure also shows less utilization of workplace-based assessment which is known for having feedback as a primary feature. The minimal use of assessment tools that are proven to assess clinical reasoning (and thus suitable for deep learning) shows less regard for FA as an approach to enhance deep learning. Reflecting the lack of understanding of FA with the consequent neglect of its most important trait: giving feedback.

### Challenges of implementing FA and suggested solutions: lack of understanding

The fragile commitment towards FA from both the medical teachers and the students is attributed to their lack of understanding of the nature of FA rather than resistance. Resistance was manifested in round one of the Delphi study when the participants generated statements such as “lack of time” and “implementation of formative assessment is exhausting”. These statements later were dropped due to non-agreement between the participant. Instead, statements related to the lack of understanding subsequently dominated the second round of the Delphi study.

Faculty development was presented as a suggested solution. The issue of enhancing the understanding of medical teachers of FA was indicated by many authors. It has been reported that clinical teachers have little knowledge about using some assessment methods as workplace-based assessment in the context of assessment for learning [17]. also, they perceive the process of giving feedback as difficult and complicated [38]. Our results shed the light on an educational culture that neglects FA and relies on summative assessment, this was also noted in other parts of the world such as Saudi Arabia [7], India [39], Malaysia [4], and Pakistan, in which even the few institutes that employ FA do not emphasize on feedback as to its major and crucial part [40].

The same issue of the lack of understanding of FA applies to the students and affects how they respond to and expect from FA. An example of this is perceiving FA as less of an important issue since its marking is not included or summed with the scores of the final exam. Subsequently, the students will be less encouraged to actively participate in the process [15]. Another example is that the students may not perceive the FA as an opportunity for learning, [38]. Furthermore, cultural and educational traditions impose a challenge to the proper implementation of FA. Although this study doesn’t represent evidence in the Sudanese context, it was reported previously in different contexts. The “culture of shy” that suppresses students from active participation plays an important role [7]. The challenges of navigating within a multicultural setting might concern the medical teacher especially if there is “negative” feedback provided as part of FA [21].

Thus, fostering FA requires different strategies. This includes providing an enabling environment, advocacy, training of the staff as well as the students, and implanting FA in the curriculum. Faculty development was also recommended by Harrison, Konings [41] and Konopasek, Norcini [6]. In our context, the authors believe that raising awareness about the value of FA among faculty and students as well will significantly improve implementation by shifting the focus from assessment of learning to assessment for learning.

### Challenges of implementing FA and suggested solutions: logistics and resources

The second theme that emerged from analyzing the qualitative assessment was the lack of resources. In the context of the investigated medical schools in this study, the issue of student-staff ratio was featured as a challenge and as an issue to be addressed among the suggested solutions. However, this issue was raised by other studies which indicate challenges such as logistic difficulties related to the number of students, technological aspects, as well as time constraints [39]. In Sudan, many medical schools suffer from a low faculty/student ratio, which in turn puts an overload on the faculty making them unable to find time for FA. The authors consider FA is not adequately addressed in many medical curricula in the country.

Resource allocations require allocating time for FA in the timetable. The use of technology and formative e-assessment is a recommendation of the current study as well as other studies [5, 11, 42]. E-assessment can utilize social media, and thus provide an opportunity for FA to be conducted at ease without the restraints of a tight timetable. Accrediting bodies also have their role by including FA as part of the requirements of the accreditation process [40]. We believe addressing FA with little or no focus on the medical curricula is an issue that needs attention. Part of allocating resources is emphasizing FA in the curricula.

## Conclusion

Fostering FA is not an easy task, it faces challenges based on the culture of “assessment of learning” and includes the lack of understanding of FA as well as the lack of resources. The medical teachers' perception and practice of FA are in desperate need of improvement. Implementation of FA requires a strategy of three approaches: faculty development, managing the curriculum by allocating time and resources for FA, and advocacy among stakeholders.

## Limitations

Although the included schools are among the largest across the country (in terms of staff number), the variation of the type of curricula and the type of medical schools was considered. But the results from this study are confined to the medical schools included in it. And since only a third of the participants were engaged in “regular” conduction of FA, the results regarding practices such as “the selection of assessment tools”, “providing feedback” and “adding the scores obtained by the student in the FA to his/her final grades of the end of the course/semester/year” are confined to this third. However, it doesn’t affect the perception of FA of the whole participants. FA tools presented in this study are limited and there is a potential for generating more options through other tools like observation or interviews in any similar future studies.

While we anticipate that an extended investigation that includes all the medical schools will yield the same conclusion, this expectation remains to be validated before generalizability is assumed.

## Supplementary Information

**Additional file 1.**

## Data Availability

All data generated or analyzed during this study are included in this published article [and its supplementary information files].

## References

[1] Gasmalla HEE. Basic Concepts. Written Assessment in Medical Education. 2023. p. 1–15.

[2] Fahal AH (2007). Medical education in the Sudan: its strengths and weaknesses. Med Teach.

[3] Sudan Medical Council (SMC) awarded Recognition Status [press release]. 2018.

[4] Ibrahim MS, Yusof MSB, Rahim AFA (2021). Why assessment which carries no grades and marks is the key for the future of education?. Educ Med J.

[5] Kibble JD, Johnson TR, Khalil MK, Nelson LD, Riggs GH, Borrero JL (2011). Insights gained from the analysis of performance and participation in online formative assessment. Teach Learn Med.

[6] Konopasek L, Norcini J, Krupat E (2016). Focusing on the formative: building an assessment system aimed at student growth and development. Acad Med.

[7] Al-Wassia R, Hamed O, Al-Wassia H, Alafari R, Jamjoom R (2015). Cultural challenges to implementation of formative assessment in Saudi Arabia: an exploratory study. Med Teach.

[8] Krasne S, Wimmers PF, Relan A, Drake TA (2006). Differential effects of two types of formative assessment in predicting performance of first-year medical students. Adv Health Sci Educ.

[9] Dannefer EF (2013). Beyond assessment of learning toward assessment for learning: educating tomorrow's physicians. Med Teach.

[10] Rahman SA (2001). Promoting learning outcomes in paediatrics through formative assessment. Med Teach.

[11] Velan GM, Jones P, McNeil HP, Kumar RK (2008). Integrated online formative assessments in the biomedical sciences for medical students: benefits for learning. BMC Med Educ.

[12] van de Ridder JM, Stokking KM, McGaghie WC, ten Cate OT (2008). What is feedback in clinical education?. Med Educ.

[13] Rushton A (2005). Formative assessment: a key to deep learning?. Med Teach.

[14] McNulty JA, Espiritu BR, Hoyt AE, Ensminger DC, Chandrasekhar AJ (2015). Associations between formative practice quizzes and summative examination outcomes in a medical anatomy course. Anat Sci Educ.

[15] Carrillo-de-la-Pena MT, Bailles E, Caseras X, Martinez A, Ortet G, Perez J (2009). Formative assessment and academic achievement in pre-graduate students of health sciences. Adv Health Sci Educ Theory Pract.

[16] Dijksterhuis MG, Schuwirth LW, Braat DD, Teunissen PW, Scheele F (2013). A qualitative study on trainees’ and supervisors’ perceptions of assessment for learning in postgraduate medical education. Med Teach.

[17] Andreassen P, Malling B (2019). How are formative assessment methods used in the clinical setting? a qualitative study. Int J Med Educ.

[18] Close B. Faculty perceptions of formative assessment and implementation practices in preclinical medical education: AQ Method Study: Oklahoma State University; 2017.

[19] AlAskari A, Alsharif A, AlSairafi A, Abbad KA, Mohamed E, Almulhem M, et al. Faculty members’ perception, implementation, and challenges of formative assessment in undergraduate medical education: a cross-sectional study. 2022. 10.21203/rs.3.rs-2285703/v1. 10.7759/cureus.74107 PMC11578639 39568485

[20] Perera J, Lee N, Win K, Perera J, Wijesuriya L (2008). Formative feedback to students: the mismatch between faculty perceptions and student expectations. Med Teach.

[21] Abraham RM, Singaram VS (2016). Third-year medical students’ and clinical teachers’ perceptions of formative assessment feedback in the simulated clinical setting. Afr J Health Prof Educ.

[22] Lajane H, Gouifrane R, Qaisar R, Chemsi G, Radid M. Perceptions, practices, and challenges of formative assessment in initial nursing education. Open J Nurs 2020;14(1):180–9.

[23] Schifferdecker KE, Reed VA (2009). Using mixed methods research in medical education: basic guidelines for researchers. Med Educ.

[24] Scheaffer RL, Mendenhall III W, Ott RL, Gerow KG. Elementary survey sampling: Cengage Learning; 2011.

[25] Yusoff MSB (2019). ABC of content validation and content validity index calculation. Educ Med J.

[26] Yusoff MSB, Arifin WN, Hadie SNH. ABC of questionnaire development and validation for survey research. Educ Med J. 2021;13(1)97-108.

[27] Hasson F, Keeney S, McKenna H (2000). Research guidelines for the Delphi survey technique. J Adv Nurs.

[28] Rana J, Sullivan A, Brett M, Weinstein AR, Atkins KM, Group SDW (2018). Defining curricular priorities for student-as-teacher programs: a national Delphi study. Med Teach.

[29] Thangaratinam S, Redman CW (2005). The delphi technique. Obstet Gynaecol.

[30] Humphrey-Murto S, Wood TJ, Gonsalves C, Mascioli K, Varpio L (2020). The Delphi Method. Acad Med.

[31] Gasmalla HEE, Mohamed Tahir MEM. A-Type MCQs. In Written Assessment in Medical Education (pp. 73-89). Cham: Springer International Publishing; 2023.

[32] Gasmalla HEE, Mohamed Tahir MEM. R-Type MCQs (Extended Matching Questions). In Written Assessment in Medical Education (pp. 91-99). Cham: Springer International Publishing; 2023.

[33] Burton JL (2009). How to write and how to answer EMQs. Obstet Gynaecol Reprod Med.

[34] Nazim SM, Talati JJ, Pinjani S, Biyabani SR, Ather MH, Norcini JJ (2019). Assessing clinical reasoning skills using Script Concordance Test (SCT) and extended matching questions (EMQs): A pilot for urology trainees. J Adv Med Educ Prof.

[35] Tan K, Chin HX, Yau CWL, Lim ECH, Samarasekera D, Ponnamperuma G (2018). Evaluating a bedside tool for neuroanatomical localization with extended-matching questions. Anat Sci Educ.

[36] Wass V, McGibbon D, Van der Vleuten C (2001). Composite undergraduate clinical examinations: how should the components be combined to maximize reliability?. Med Educ.

[37] Wilson R, Case S. Extended matching questions: an alternative to multiple-choice or free-response questions. J Vet Med Educ. 1993;20(3).

[38] Harrison CJ, Könings KD, Dannefer EF, Schuwirth LW, Wass V, van der Vleuten CP (2016). Factors influencing students’ receptivity to formative feedback emerging from different assessment cultures. Perspect Med Educ.

[39] Sharma S, Sharma V, Sharma M, Awasthi B, Chaudhary S (2015). Formative assessment in postgraduate medical education - Perceptions of students and teachers. Int J Appl Basic Med Res.

[40] Rauf A, Shamim MS, Aly SM, Chundrigar T, Alam SN (2014). Formative assessment in undergraduate medical education: concept, implementation and hurdles. J Pak Med Assoc.

[41] Harrison CJ, Konings KD, Schuwirth LWT, Wass V, van der Vleuten CPM (2017). Changing the culture of assessment: the dominance of the summative assessment paradigm. BMC Med Educ.

[42] Johan Krumsvik R, Ludvigsen K (2013). Theoretical and methodological issues of formative e-assessment in plenary lectures. Int J Pedagog Learn.

